# *N*-(4-Chloro­phen­yl)-4-methyl­pyridin-2-amine

**DOI:** 10.1107/S1600536810030138

**Published:** 2010-07-31

**Authors:** Zainal A. Fairuz, Zaharah Aiyub, Zanariah Abdullah, Seik Weng Ng, Edward R. T. Tiekink

**Affiliations:** a Department of Chemistry, University of Malaya, 50603 Kuala Lumpur, Malaysia

## Abstract

In the title compound, C_12_H_11_ClN_2_, the dihedral angle between the benzene and pyridyl rings is 48.03 (8)°. Twists are also evident in the mol­ecule, in particular about the N_a_–C_b_ (a = amine and b = benzene) bond [C—N—C—C = −144.79 (18)°]. In the crystal, inversion dimers linked by pairs of N—H⋯N hydrogen bonds result in the formation of eight-membered {⋯NCNH}_2_ synthons [or *R*_2_^2^(8) loops].

## Related literature

For background to the fluorescence properties of compounds related to the title compound, see: Kawai *et al.* (2001 ▶); Abdullah (2005 ▶).
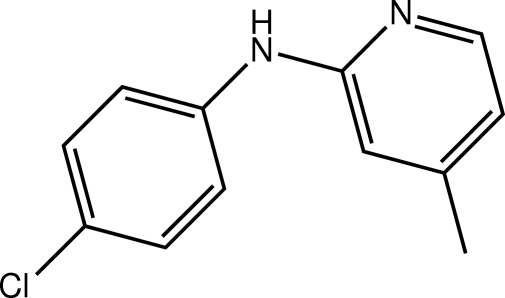


## Experimental

### 

#### Crystal data

C_12_H_11_ClN_2_
*M* *_r_* = 218.68
Monoclinic, 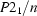

*a* = 15.9335 (15) Å
*b* = 4.0651 (4) Å
*c* = 17.0153 (16) Å
β = 98.755 (1)°
*V* = 1089.26 (18) Å^3^
*Z* = 4
Mo *K*α radiation
μ = 0.32 mm^−1^
*T* = 293 K
0.30 × 0.30 × 0.20 mm

#### Data collection

Bruker SMART APEX CCD diffractometer
Absorption correction: multi-scan (*SADABS*; Sheldrick, 1996 ▶) *T*_min_ = 0.776, *T*_max_ = 0.862
9785 measured reflections
2509 independent reflections
1886 reflections with *I* > 2σ(*I*)
*R*_int_ = 0.030

#### Refinement

*R*[*F* ^2^ > 2σ(*F* ^2^)] = 0.042
*wR*(*F* ^2^) = 0.132
*S* = 1.04
2509 reflections
141 parameters
1 restraint
H atoms treated by a mixture of independent and constrained refinement
Δρ_max_ = 0.22 e Å^−3^
Δρ_min_ = −0.18 e Å^−3^

### 

Data collection: *APEX2* (Bruker, 2009 ▶); cell refinement: *SAINT* (Bruker, 2009 ▶); data reduction: *SAINT*; program(s) used to solve structure: *SHELXS97* (Sheldrick, 2008 ▶); program(s) used to refine structure: *SHELXL97* (Sheldrick, 2008 ▶); molecular graphics: *ORTEP-3* (Farrugia, 1997 ▶) and *DIAMOND* (Brandenburg, 2006 ▶); software used to prepare material for publication: *publCIF* (Westrip, 2010 ▶).

## Supplementary Material

Crystal structure: contains datablocks global, I. DOI: 10.1107/S1600536810030138/hb5582sup1.cif

Structure factors: contains datablocks I. DOI: 10.1107/S1600536810030138/hb5582Isup2.hkl

Additional supplementary materials:  crystallographic information; 3D view; checkCIF report

## Figures and Tables

**Table 1:** Hydrogen-bond geometry (Å, °)

*D*—H⋯*A*	*D*—H	H⋯*A*	*D*⋯*A*	*D*—H⋯*A*
N2—H2*n*⋯N1^i^	0.86 (1)	2.19 (1)	3.029 (2)	167 (2)

Symmetry code: (i) 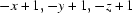.

**Fig. 1.**
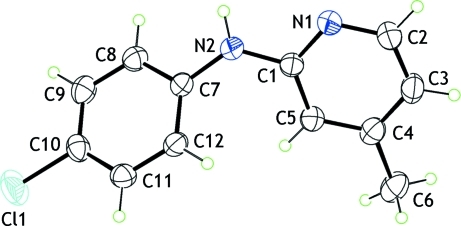
The molecular structure of (I) showing displacement ellipsoids at the 35% probability level.

**Fig. 2.**
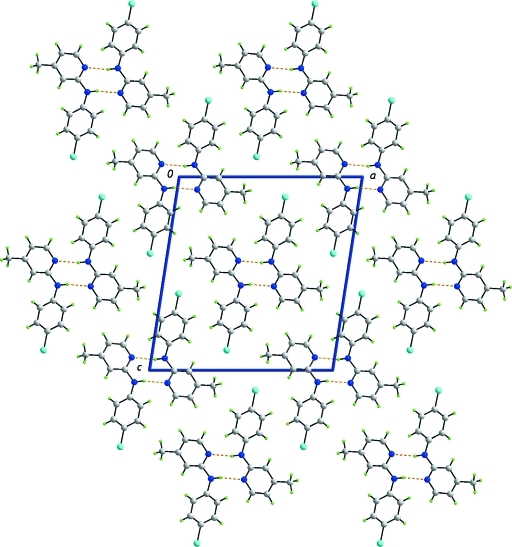
Unit-cell contents shown in projection down the b axis in (I). The N–H···N hydrogen bonding is shown as orange dashed lines.

## supplementary crystallographic information

### Comment

The title compound, (I), was investigated in the context of potential fluorescence properties (Kawai *et al.* 2001; Abdullah, 2005). The molecular structure of (I), Fig. 1, shows that the molecule is non-planar as seen in the dihedral angle of 48.03 (8) ° formed between the benzene and pyridyl rings, and in the twists about the central N–C bonds, *i.e*. the C7–N2–C1–N1 and C1–N2–C7–C8 torsion angles are -167.92 (17) and -144.79 (18) °, respectively. The amine-H and pyridine-N atoms are orientated in the same direction, an arrangement that facilitates the formation of N–H···N hydrogen bonds. Thus, centrosymmetrically related molecules are linked *via* N–H···N hydrogen bonds that lead to eight-membered {···NCNH}_2_ synthons, Table 1. The dimeric aggregates stack along the *b* axis, Fig. 2.

### Experimental

2-Chloro-4-methylpyridine (1.0 ml, 1.14 mmol) was added to 4-chloroaniline (1.4543 g, 1.14 mmol) and heated for 2 h. The mixture was cooled and dissolved water (15 ml), extracted with diethyl ether (3 × 10 ml), washed with water (3 × 10 ml), and then dried over anhydrous sodium sulfate. Evaporation of the solvent gave a gray solid. Recrystallization from ethanol yielded colourless blocks of (I).

### Refinement

Carbon-bound H-atoms were placed in calculated positions (C—H 0.93 to 0.96 Å) and were included in the refinement in the riding model approximation, with *U*_iso_(H) set to 1.2 to 1.5*U*_equiv_(C). The N-bound H-atom was located in a difference Fourier map, and was refined with a distance restraint of N–H 0.86±0.01 Å; the *U_iso_* value was freely refined.

### Crystal data

**Table:**

C_12_H_11_ClN_2_	*F*(000) = 456
*M**_r_* = 218.68	*D*_x_ = 1.333 Mg m^−^^3^
Monoclinic, *P*2_1_/*n*	Mo *K*α radiation, λ = 0.71073 Å
Hall symbol: -P 2yn	Cell parameters from 2763 reflections
*a* = 15.9335 (15) Å	θ = 2.4–25.7°
*b* = 4.0651 (4) Å	µ = 0.32 mm^−^^1^
*c* = 17.0153 (16) Å	*T* = 293 K
β = 98.755 (1)°	Block, colourless
*V* = 1089.26 (18) Å^3^	0.30 × 0.30 × 0.20 mm
*Z* = 4	

### Data collection

**Table:**

Bruker SMART APEX CCD diffractometer	2509 independent reflections
Radiation source: fine-focus sealed tube	1886 reflections with *I* > 2σ(*I*)
graphite	*R*_int_ = 0.030
ω scans	θ_max_ = 27.5°, θ_min_ = 1.9°
Absorption correction: multi-scan (*SADABS*; Sheldrick, 1996)	*h* = −20→19
*T*_min_ = 0.776, *T*_max_ = 0.862	*k* = −5→5
9785 measured reflections	*l* = −22→20

### Refinement

**Table:**

Refinement on *F*^2^	Primary atom site location: structure-invariant direct methods
Least-squares matrix: full	Secondary atom site location: difference Fourier map
*R*[*F*^2^ > 2σ(*F*^2^)] = 0.042	Hydrogen site location: inferred from neighbouring sites
*wR*(*F*^2^) = 0.132	H atoms treated by a mixture of independent and constrained refinement
*S* = 1.04	*w* = 1/[σ^2^(*F*_o_^2^) + (0.0689*P*)^2^ + 0.1992*P*] where *P* = (*F*_o_^2^ + 2*F*_c_^2^)/3
2509 reflections	(Δ/σ)_max_ < 0.001
141 parameters	Δρ_max_ = 0.22 e Å^−^^3^
1 restraint	Δρ_min_ = −0.18 e Å^−^^3^

### Special details

**Table:**

Geometry. All e.s.d.'s (except the e.s.d. in the dihedral angle between two l.s. planes) are estimated using the full covariance matrix. The cell e.s.d.'s are taken into account individually in the estimation of e.s.d.'s in distances, angles and torsion angles; correlations between e.s.d.'s in cell parameters are only used when they are defined by crystal symmetry. An approximate (isotropic) treatment of cell e.s.d.'s is used for estimating e.s.d.'s involving l.s. planes.
Refinement. Refinement of *F*^2^ against ALL reflections. The weighted *R*-factor *wR* and goodness of fit *S* are based on *F*^2^, conventional *R*-factors *R* are based on *F*, with *F* set to zero for negative *F*^2^. The threshold expression of *F*^2^ > σ(*F*^2^) is used only for calculating *R*-factors(gt) *etc*. and is not relevant to the choice of reflections for refinement. *R*-factors based on *F*^2^ are statistically about twice as large as those based on *F*, and *R*- factors based on ALL data will be even larger.

### Fractional atomic coordinates and isotropic or equivalent isotropic displacement parameters (Å^2^)

**Table:**

	*x*	*y*	*z*	*U*_iso_*/*U*_eq_	
Cl1	0.59502 (4)	1.02913 (16)	0.10424 (3)	0.0834 (2)	
N1	0.60831 (9)	0.5290 (4)	0.56614 (8)	0.0495 (4)	
N2	0.57324 (9)	0.6997 (4)	0.43820 (9)	0.0552 (4)	
H2n	0.5222 (7)	0.653 (5)	0.4444 (11)	0.061 (6)*	
C1	0.63451 (10)	0.6751 (4)	0.50365 (9)	0.0439 (4)	
C2	0.66466 (12)	0.5088 (5)	0.63260 (11)	0.0578 (5)	
H2	0.6472	0.4101	0.6767	0.069*	
C3	0.74612 (12)	0.6230 (5)	0.64035 (10)	0.0570 (5)	
H3	0.7827	0.5985	0.6881	0.068*	
C4	0.77369 (11)	0.7769 (4)	0.57556 (10)	0.0498 (4)	
C5	0.71648 (10)	0.8019 (4)	0.50667 (10)	0.0461 (4)	
H5	0.7323	0.9030	0.4621	0.055*	
C6	0.86175 (12)	0.9137 (6)	0.58031 (13)	0.0661 (5)	
H6A	0.8620	1.0847	0.5414	0.099*	
H6B	0.8999	0.7416	0.5701	0.099*	
H6C	0.8797	1.0023	0.6325	0.099*	
C7	0.58319 (10)	0.7850 (4)	0.36041 (9)	0.0428 (4)	
C8	0.51945 (11)	0.9664 (4)	0.31600 (11)	0.0496 (4)	
H8	0.4740	1.0406	0.3396	0.059*	
C9	0.52239 (12)	1.0389 (4)	0.23723 (11)	0.0544 (4)	
H9	0.4789	1.1588	0.2077	0.065*	
C10	0.59014 (12)	0.9323 (4)	0.20290 (10)	0.0493 (4)	
C11	0.65418 (11)	0.7545 (4)	0.24569 (10)	0.0481 (4)	
H11	0.6998	0.6843	0.2219	0.058*	
C12	0.65108 (10)	0.6791 (4)	0.32428 (10)	0.0462 (4)	
H12	0.6946	0.5570	0.3532	0.055*	

### Atomic displacement parameters (Å^2^)

**Table:**

	*U*^11^	*U*^22^	*U*^33^	*U*^12^	*U*^13^	*U*^23^
Cl1	0.1035 (5)	0.0997 (5)	0.0472 (3)	−0.0123 (3)	0.0123 (3)	0.0172 (3)
N1	0.0440 (8)	0.0626 (9)	0.0425 (8)	0.0058 (6)	0.0082 (6)	0.0026 (6)
N2	0.0364 (8)	0.0858 (12)	0.0428 (8)	−0.0052 (7)	0.0048 (6)	0.0083 (7)
C1	0.0414 (8)	0.0494 (9)	0.0409 (8)	0.0059 (7)	0.0061 (6)	−0.0034 (7)
C2	0.0582 (11)	0.0722 (13)	0.0430 (9)	0.0070 (9)	0.0072 (8)	0.0058 (8)
C3	0.0574 (11)	0.0673 (11)	0.0424 (9)	0.0083 (9)	−0.0051 (8)	−0.0042 (8)
C4	0.0481 (9)	0.0476 (9)	0.0515 (10)	0.0039 (7)	0.0006 (7)	−0.0127 (7)
C5	0.0452 (9)	0.0486 (9)	0.0439 (9)	−0.0008 (7)	0.0049 (7)	−0.0019 (7)
C6	0.0535 (11)	0.0693 (12)	0.0705 (13)	−0.0074 (9)	−0.0066 (9)	−0.0119 (10)
C7	0.0379 (8)	0.0485 (9)	0.0409 (8)	−0.0039 (7)	0.0027 (6)	−0.0004 (7)
C8	0.0436 (9)	0.0535 (10)	0.0512 (10)	0.0057 (7)	0.0059 (7)	−0.0012 (7)
C9	0.0539 (10)	0.0521 (10)	0.0541 (10)	0.0051 (8)	−0.0019 (8)	0.0089 (8)
C10	0.0572 (10)	0.0488 (9)	0.0411 (8)	−0.0110 (8)	0.0052 (7)	0.0025 (7)
C11	0.0440 (9)	0.0518 (10)	0.0495 (9)	−0.0053 (7)	0.0101 (7)	−0.0027 (7)
C12	0.0373 (8)	0.0517 (9)	0.0486 (9)	0.0024 (7)	0.0032 (7)	0.0034 (7)

### Geometric parameters (Å, °)

**Table:**

Cl1—C10	1.7376 (18)	C6—H6A	0.9600
N1—C2	1.335 (2)	C6—H6B	0.9600
N1—C1	1.339 (2)	C6—H6C	0.9600
N2—C1	1.368 (2)	C7—C8	1.383 (2)
N2—C7	1.400 (2)	C7—C12	1.391 (2)
N2—H2n	0.857 (9)	C8—C9	1.380 (2)
C1—C5	1.398 (2)	C8—H8	0.9300
C2—C3	1.366 (3)	C9—C10	1.373 (3)
C2—H2	0.9300	C9—H9	0.9300
C3—C4	1.396 (3)	C10—C11	1.367 (2)
C3—H3	0.9300	C11—C12	1.380 (2)
C4—C5	1.375 (2)	C11—H11	0.9300
C4—C6	1.500 (3)	C12—H12	0.9300
C5—H5	0.9300		
			
C2—N1—C1	116.69 (15)	C4—C6—H6C	109.5
C1—N2—C7	128.17 (14)	H6A—C6—H6C	109.5
C1—N2—H2n	117.2 (13)	H6B—C6—H6C	109.5
C7—N2—H2n	114.6 (13)	C8—C7—C12	118.64 (15)
N1—C1—N2	114.12 (15)	C8—C7—N2	117.97 (15)
N1—C1—C5	122.47 (15)	C12—C7—N2	123.27 (15)
N2—C1—C5	123.36 (15)	C9—C8—C7	120.91 (16)
N1—C2—C3	124.59 (18)	C9—C8—H8	119.5
N1—C2—H2	117.7	C7—C8—H8	119.5
C3—C2—H2	117.7	C10—C9—C8	119.40 (16)
C2—C3—C4	119.00 (16)	C10—C9—H9	120.3
C2—C3—H3	120.5	C8—C9—H9	120.3
C4—C3—H3	120.5	C11—C10—C9	120.77 (16)
C5—C4—C3	117.32 (16)	C11—C10—Cl1	119.67 (14)
C5—C4—C6	120.81 (17)	C9—C10—Cl1	119.55 (14)
C3—C4—C6	121.87 (16)	C10—C11—C12	119.99 (16)
C4—C5—C1	119.92 (16)	C10—C11—H11	120.0
C4—C5—H5	120.0	C12—C11—H11	120.0
C1—C5—H5	120.0	C11—C12—C7	120.28 (15)
C4—C6—H6A	109.5	C11—C12—H12	119.9
C4—C6—H6B	109.5	C7—C12—H12	119.9
H6A—C6—H6B	109.5		
			
C2—N1—C1—N2	−177.70 (15)	C1—N2—C7—C8	−144.79 (18)
C2—N1—C1—C5	0.0 (2)	C1—N2—C7—C12	39.4 (3)
C7—N2—C1—N1	−167.92 (17)	C12—C7—C8—C9	0.6 (3)
C7—N2—C1—C5	14.4 (3)	N2—C7—C8—C9	−175.42 (16)
C1—N1—C2—C3	−0.7 (3)	C7—C8—C9—C10	−0.8 (3)
N1—C2—C3—C4	1.0 (3)	C8—C9—C10—C11	0.3 (3)
C2—C3—C4—C5	−0.6 (3)	C8—C9—C10—Cl1	−178.88 (14)
C2—C3—C4—C6	178.85 (18)	C9—C10—C11—C12	0.2 (3)
C3—C4—C5—C1	−0.1 (2)	Cl1—C10—C11—C12	179.44 (13)
C6—C4—C5—C1	−179.50 (17)	C10—C11—C12—C7	−0.4 (3)
N1—C1—C5—C4	0.4 (3)	C8—C7—C12—C11	−0.1 (2)
N2—C1—C5—C4	177.85 (16)	N2—C7—C12—C11	175.77 (15)

### Hydrogen-bond geometry (Å, °)

**Table:**

*D*—H···*A*	*D*—H	H···*A*	*D*···*A*	*D*—H···*A*
N2—H2n···N1^i^	0.86 (1)	2.19 (1)	3.029 (2)	167 (2)

Symmetry codes: (i) −*x*+1, −*y*+1, −*z*+1.
